# Supplementary material for: Functional visualization of NK cell-mediated killing of metastatic single tumor cells
Source: eLife. 2022 Feb 3;11:e76269. doi: 10.7554/eLife.76269 (PMC8849286; doi:10.7554/eLife.76269)
Supplement: Source code 1. [file elife-76269-code1.zip › MATLAB_code.pdf]

Fitting_1b; Figure 1B

Main_191017.m; Figure 3

- Functions associated with Main_191017.m
  - VisualizeTrajectory(ncell, indx)
  - SetPositionToOrigin(cell)
  - ReplaceZeroToNan(cell, maxt_list)
  - ReplaceMissingTimeToNan(cell, maxt_list)
  - ImportAndConvertTable(id, flag)
  - GraphMSD(msd0, msd1)
  - GraphInstSpeed(inst_speed0, inst_speed1)
  - ConvertToTable(data)
  - CellMigrationQuantification(cell)
  - CalculationHitProbability(cnt_in_all, maxt_list_c_all)
  - HitCancer(nk, cancer, inner, outer)
  - RearrangeTime(cell)

Lung_NK.m; Supplementary Table
